# Recent Views on Tuberculosis

**Published:** 1908-11-14

**Authors:** 


### The Hospital

A Journal of

The Medical Sciences and Hospital Administration.

New Series. No. 88, Vol. IV. [No. 1160, Vol. XLV.] SATURDAY, NOVEMBER 14, 1908.

### Recent Views on Tuberculosis.

Most of our readers will remember well the agitation caused by Dr. Koch when at the Tuberculosis Congress held in London in 1901 he enunciated his belief that to all intents and purposes bovine tuberculosis is not communicable to man. His actual conclusions were as follows: That the bacilli of bovine tuberculosis are different from those of human tuberculosis; that human beings may be infected by bovine tubercle bacilli, but that serious diseases from this cause occur very rarely; that, in consequence, preventive measures against tuberculosis should be directed primarily against the propagation of human tubercle bacilli. Seven years have passed; the question has been thrashed out in almost every civilised country, and has been the subject of a Royal Commission in these islands. Gradually the battle, which raged so furiously immediately after the pronouncement of the great bacteriologist, has died away, as the results of animal experiment and post-mortem observation accumulated. Finally, when it appeared that the struggle was over, since no one could be found to support the thesis of Professor Koch, we find the Professor himself returning to the charge at the International Congress at Washington, and re-affirming his conviction that he was right in 1901; that with few exceptions the bacilli of bovine tuberculosis are but slightly virulent to man, and tend to remain localised, and that the few reported cases in which the bovine bacilli have produced a progressive and fatal general tuberculosis in man are not above suspicion. It would seem that Professor Koch stood quite alone, and several of the succeeding speakers referred in strong terms to the gravity of assuming that the bovine disease cannot be transmitted to man except as a rarity and in a mitigated form. "I would consider it an extreme misfortune," Dr. Ravenel is reported to have said, "not only for this country but for every country on the face of the earth if any impression should go from this meeting that the deaths due to the bovine bacillus are a negligible quantity." There are few people we fancy, certainly among those to whom it has fallen to perform post-mortem examinations at hospitals for children, who will not subscribe to Dr. Ravenel's belief; and there is something rather pathetic in the spectacle of the first bacteriologist of the day thus standing at bay against an almost universal consensus of opinion.

At a later sitting of the Congress Professor Calmette communicated some further statistics regarding the value of the conjunctival tuberculin reaction which goes by his name. Of 2,894 patients clinically tuberculous, 92.05 per cent. gave positive reactions to the conjunctival test, while of 2,328 persons who were without suspicion of tuberculosis, 16.8 per cent. gave similar positive reactions. Of 55 subjects who, having reacted positively to the conjunctival test, although clinically free from suspicion of tuberculosis, came subsequently to the post-mortem room, 49 showed macroscopic tuberculous lesions, most often in the lymphatic glands at the bifurcation of the trachea. In a series of more than 6,000 tests the ophthalmic complications were as follows: Three cases of phlyctenular keratitis, twenty of conjunctivitis, and "seventy-two instaaces in which the reaction was prolonged beyond three weeks." In no case did graver ocular disturbance or permanent visual defect attend the use of the test. As a general rule the reaction in cases of suspected tuberculosis occurred early, being both later in its appearance and slighter in its manifestations when the tuberculous lesions were well developed. Patients the subjects of advanced tuberculosis reacted hardly at all.

Since Professor Calmette announced his method of diagnosing tuberculosis by instillation of tuberculin into the eye there has been no little disagreement, not only concerning the value of the test but also concerning the dangers attendant upon it. The reaction consists, it will be remembered, in an inflammatory response of the conjunctiva which it is contended is peculiar to tuberculous subjects. In such patients within three to six hours of the instillation the eye becomes red and exhibits some congestion of the lids. The subjective symptoms are slight as a rule, and the eye recovers within two or three days if the reaction takes a normal course. From time to time, however, the response has been so active as to inflict lasting damage upon the eye. This, of course, is so disconcerting a possibility that the value of the test in practice must stand or fall by it. Apart altogether from anything so catastrophic as permanent impairment of vision, it is a very arguable proposition whether approximate certainty of diagnosis is not dearly bought at the expense of three or four weeks of serious conjunctivitis. There seems to be little doubt that more or less violent reactions have to be looked for from time to time, and it would be interesting to know to what cause we are to attribute the prolongation of the reaction to a period of three weeks and upwards in Professor Calmette's series. Here he speaks of seventy-two instances in which this prolongation took place, yet quotes them apart from twenty instances of admitted conjunctivitis. In a paper communicated to the last meeting of the British Medical Association Dr. J. H. Sequeira speaks in high terms of the value of the reaction in the diagnosis of skin diseases. "The results," he says, "are quite satisfactory. No case of tuberculous disease of the skin failed to give a positive reaction. In certain cases a positive reaction helped to form a diagnosis; in others a negative reaction has been of equal value. . . . If the eyes are normal there does not appear to be any risk in applying a 0.5 solution, provided the solution be freshly made." On the whole it seems that the test is reliable in a majority of cases, and where a positive diagnosis is held to be of paramount importance the possible dangers of the test are not sufficient, in careful hands, to preclude the operation. None the less there is good ground for thinking that the routine use of this reaction does not give a return compensatory of the discomfort and risk occasionally attending it.

